# Spatiotemporal characteristics and influencing factors of network attention to resort hotels in China

**DOI:** 10.1016/j.heliyon.2024.e35314

**Published:** 2024-07-26

**Authors:** Huazhen Sun, Yifeng Zhang, Weifeng Guo

**Affiliations:** a School of Tourism, Wuyi University, Nanping, 354300, China; b Graduate school of business, SEGi University, Kota Damansara, 47810, Malaysia

**Keywords:** Resort hotels, Network attention, Spatial and temporal characteristics, Influencing factors, Baidu index

## Abstract

An increasing number of people are gathering travel information online prior to their trips as a result of the Internet's rapid expansion. The amount of network attention receives can indicate how many people are looking for something. The orderly development of Chinese resort hotels can be guided by research on the spatiotemporal characteristics of their network attention. Using Chinese resort hotels as the study subject, everyday information about resort hotels in 31 Chinese provinces was gathered via the Baidu Index platform between 2018 and 2022, and mathematical statistics and other methods were used to study the spatiotemporal distribution characteristics and influence of Chinese resort hotels network attention. Findings reveal that, from 2018 to 2022, network attention to resort hotels across the country fluctuated significantly across seasons, and there was a “precursor effect” reaction before the week of network attention. Moreover, the spatial distribution of network attention to Chinese resort hotels was uneven, showing an overall trend of “east–central–west” decline. Level of economic development, degree of network development, leisure time, and population size are the main factors affecting the spatiotemporal distribution of Chinese resort hotels network attention.

## 1 Introduction

In recent decades, the tourist industry has experienced unparalleled expansion worldwide, making tourism demand forecasting a crucial area of research for scholars worldwide [1]. The 51st report from China Internet Information says that there were 1.067 billion Internet users in China in December 2022, which is a 75.6 % Internet usage rate. Tourists collect the travel information they want before traveling, and the Internet is one of the quickest and most convenient channels to do so [[2], [3], [4], [5], [6]]. Tourists' search records form big data that help other travelers looking for similar information. The value of the search volume of the keywords in the search engine platform reflects the network attention index. The primary search engines utilized by Chinese users are Baidu, Bing, and Google. Baidu, in particular, holds a dominant share of around 72.4 % of search queries [7]. Hence, the Baidu Index is one of the big data indicators used to study network attention and has, in recent years, been among the search platforms used by many scholars in the field of tourism as a way to study the behavioral characteristics and influencing factors of tourists’ searches [7,8]. In the current era of big data, the behavior of visitors demonstrates a shift towards intelligence and personalization. Therefore, it is crucial to analyses their spatiotemporal behavior using network data in order to understand their preferences and enhance the appeal of hotels.

Chinese people's living standards are rising as China's social economy develops quickly, and more and more new consumer demands are emerging [8]. The basic functions provided by hotels in the past are no longer able to satisfy consumers, and consumers' needs are diversifying to include, for example, healthcare, vacations, and leisure activities. The resort hotels can meet the diversified needs of consumers and provide comprehensive services, such as catering, accommodation, leisure activities, and entertainment for vacationers. Resort hotels used to be synonymous with “high-end” and “luxury”, but as leisure and vacation travel has begun to boom, more and more tourists travel to play would like to choice resort hotels. But now that more and more resort hotels are opening, how to attract tourists in a competitive market has become a topic worth studying.

Studies on resort hotels have examined oral communication, the effect of brand image, and pricing perception on consumers' purchasing decisions [9], tackling food waste [10], dynamic pricing [11], online and offline bookings [12], sensory marketing [13], and customer satisfaction and loyalty [[14], [15], [16]]. Most studies take specific resort hotels as examples, and there is limited research using big data to study resort hotels from a macro perspective, especially the behavior of resort hotels guests.

The geographical pattern of tourism flow and the distribution of tourist sites have been clarified in certain studies through the use of big data. Additionally, network attention data has been employed to develop innovative marketing strategies for scenic areas [[17], [18], [19]], and network attention has been studied in research on tourism [[20], [21], [22]] and marketing strategies [[23], [24], [25], [26]]. Although many scholars have applied big data to the tourism field, fewer studies have considered the spatiotemporal characteristics of the tourism accommodation industry from a macroscopic perspective, and only the spatiotemporal distribution characteristics of network attention to B&Bs and hotel chains have been studied. To address these research deficiencies, this work aims to take network attention as the core point of research and resort hotels in China as the scope of research, collect the data of tourists using the internet search tool to search for “resort hotels” as the keyword before travel, and analyze the spatial and temporal characteristics and influencing factors to reveal the spatial and temporal distribution pattern of demand for resort hotels in China. This study contributes to providing scientific support for the rational development of resort hotels and a basis for corporate marketing strategies. Furthermore, it offers novel perspectives on how the utilization of big data enhances the precision of short-term forecasts regarding the monthly influx of tourists in resort hotels.

## 2 Literature review

The utilization of big data was initially employed by scholars in the Western world [27,28]. They mostly used Google Trends to look into topics like search behavior and how customers make decisions [1,22,[29], [30], [31]]. A user's level of engagement with a phenomenon or object may be reflected in their search volume. Additionally, there exists a correlation between search data and the actual social conduct of consumers to a certain degree [32].

Through big data from online searches, network attention indicators, like Google Trends and the Baidu Index, can indicate the preferences of Internet users' behavior and the recognition of public opinion. Research has used big data to study information search behaviors and tourist characteristics [19], Assess the prediction accuracy of the search data from Google and Baidu [20], study cultural tourism internet attention in China [21], used GPS trajectory big data to research residents' choices of park green spaces [33], conducted an analysis of the online forums containing large amounts of data from 7 cities in Europe and offered a new model [26], compare traditional and big data information sources, provide a basis for the selection of information sources useful when looking at urban sustainable tourism research [34], utilize a comprehensive dataset from 282 Chinese prefecture-level cities spanning a period of 9 years to enhance the advancement of ecotourism [35], and use big data to research the spatiotemporal of scenic spots, analyze tourists' behavior in choosing destinations when traveling [8,[36], [37], [38], [39]]. However, limited research focus uses the big data to find the hotel tourists’ behavior.

Chinese scholars have used the Baidu Index and other search engines to predict tourist numbers, analyze tourism industry security issues, estimate passenger flow using customer data, and study the geographical distribution of tourist site concerns [8,36]. Network attention data from multiple network platforms and social media, including Sina Weibo, along with data from the Baidu Index, have been utilized to examine the origin markets of tourists. This includes analyzing the spatial network structure of tourism flow and identifying the factors that influence it, with the aim of enhancing the sustainable development of tourism [[40], [41], [42]], found the features and factors in space and time that affect how much attention is paid to the Chinese red tourist network [43], examine how the social network members of visitors impact their decision-making process while choosing destination [44]. Nevertheless, the use of the Baidu Index for investigating resort hotels in China and examining the spatiotemporal attributes and factors that impact network attention towards hotels, particularly resort hotels is limited.

## 3 Data sources and methodology

### 3.1 Data sources

The Baidu Index is a software programmer developed by Baidu that evaluates internet search data to determine the level of user and media attention for a specific topic. The Index can offer daily search volumes from users, which can indicate the level of interest people have in a specific keyword and provide demographic information based on user demand.

In this study, “resort hotels” was used as the search keyword, and the time period was set at January 1, 2018, to December 31, 2022. The daily web search volume during the five-year period was counted through the official website of Baidu Index as the basic data of the time distribution characteristics of resort hotels network attention. In order to analyze the spatial distribution of the network attention of resort hotels, the daily web search volume was calculated by taking province and region of China as spatial unit respectively.

### 3.2 Methodology

This study utilizes the approach of regional economic difference analysis to examine the level of attention given to resort hotels by network users in 31 provincial-level administrative regions in China. The analysis is based on five indicators: coefficient of variation (CV), Herfindahl coefficient (H), primacy (P), geographic concentration index (G), seasonal concentration index (S). The objective is to examine characteristics of spatial and temporal differences in the distribution of resort hotels throughout the 31 provincial-level administrative regions in China from 2018 to 2022. Each of these indicators is explained below.

(1) The CV is the ratio of the standard deviation to the mean, is utilized to analyze and evaluate the level of variation among sample indicators of economic size in various locations. The disparity between the amount of network attention given to resort hotels in each province and area of China becomes more apparent as the CV value increases [45].

$$CV=\sqrt{\frac{\sum_{i=1}^{n}{\left(x_{i}-\bar{x}\right)}^{2}}{n}}/\bar{x}$$

(2) The H is employed to assess the level of agglomeration in a regional economic scale index. A higher value of the H index indicates a greater degree of regional economic agglomeration, with a value closer to 1 indicating a higher level of agglomeration. Greater regional concentration of resort hotels' network focus is indicated by a H value that is closer to 1.

$$H=\sum_{i=1}^{n}p_{i}^{2}$$

(3) The P is employed to assess the distribution of regional economic scale, indicating the level of economic concentration in a region. The calculation involves determining the ratio between the largest and the second largest economic scale. The resort hotels network's attention becomes more concentrated and imbalanced as the P value increases [46].

$$P=p_{1}/p_{2}$$

(4) The G is employed to analyze and contrast the distribution of network attention towards resort hotels across various geographies. A higher G number indicates that there is a greater concentration of resort hotels in a specific area [47].

$$G=100\times \sqrt{\sum_{i=1}^{n}{\left(p_{j}/p\right)}^{2}}$$

(5) The S is employed to assess and contrast the temporal distribution of attention across the resort hotels network, specifically in relation to different months. A lower S value indicates less temporal variation in the attention received by resort hotels in the network, resulting in a more evenly distributed monthly distribution [48].

$$S=\sqrt{\sum_{i=1}^{12}{\left(x_{i}-8.33\right)}^{2}∕12}$$

### 3.3 Analysis of the spatial and temporal characteristics of resort hotels network attention

#### 3.3.1 Temporal differences in resort hotels network attention

From Table 1, we can see that the network attention index of resort hotels in 2018–2022 were 629,677, 619,072, 485,194, 403,337, and 379,705, respectively. Taking 2020 as the dividing point, the network attention of resort hotels in 2018–2019 shows a stable trend, while, in the period 2020–2022, it decreases sharply due to the influence of the COVID-19 pandemic, and the overall trend is decreasing. In 2018, the network attention of resort hotels fluctuated, rising from January to July, reaching a small peak in July and falling back significantly after October. In 2019, network attention formed two peaks in April and August. In 2020, network attention tended to rise gradually from January, reached a small peak in May, and showed a fluctuating downward trend from June. In 2021, the network attention of resort hotels formed two peaks in April and July and started to decline after July. In 2022, the network attention index of resort hotels reached its maximum in March and showed an overall decreasing trend throughout the year. The network attention index in 2020–2022 is obvious: both are lower than the data in 2018 and 2019.

**Table 1:** Resort hotels network attention index by month in China, 2018–2022.

Month/parameters	Year				
	2018	2019	2020	2021	2022
January	44,130	49,977	39,227	28,365	24,252
February	40,263	38,422	30,015	21,985	27,565
March	51,153	48,092	42,767	38,025	41,519
April	56,583	56,828	45,832	43,443	32,472
May	53,567	48,116	47,974	37,867	34,668
June	54,357	47,132	45,078	41,874	30,447
July	66,512	54,616	44,722	42,294	38,928
August	61,515	65,895	41,286	27,178	36,002
September	56,073	60,679	42,663	35,168	33,921
October	49,531	48,611	35,956	30,338	30,093
November	47,657	52,771	34,462	26,947	26,267
December	48,336	47,933	35,212	29,853	23,571
Seasonal concentration index	1.1069	1.1099	1.3891	1.6848	1.4278
Herfindahl Index	0.0848	0.0848	0.0847	0.0867	0.0858

The summary of the network attention index of resort hotels by months found that the network attention index of resort hotels showed a steady growth from January to April, reached a peak of annual search volume in July and August, and then gradually declined, a pattern coinciding with the traditional summer vacation peak season. Table 1 shows that the seasonal concentration index of attention towards the resort hotels' network is 1.1069, 1.1099, 1.3891, 1.6848, and 1.4278 from 2018 to 2022. These large values indicate that China's resort hotels network attention index is unbalanced in monthly distribution and has relatively large seasonal differences. It can be divided into flat, low and high seasons. Calculating and dividing the average monthly network attention for each month during the period 2018–2022, we find that March–September is the peak season, the flat season is October–December, and the low season is January–February. According to the traditional division of the four seasons, the data of the network attention of resort hotels are summarized into four quarters, for which the quarterly distribution characteristics of the network attention of resort hotels are analyzed. In the period 2018–2022, the network attention data of the four quarters of spring, summer, fall, and winter were 565,757, 676,238, 707,452, and 567,538, respectively. The data show that the summer and fall seasons had the most resort hotels network attention, which partially overlaps with the timing of the peak passenger season. The H coefficients of national resort hotels network attention from 2018 to 2022 are 0.0848, 0.0848, 0.0847, 0.0867, and 0.0858, which all tend to be close to 0, indicating that the resort hotels network attention is significantly different seasonally, and the distribution of attention is unbalanced.

**Fig. 1:**
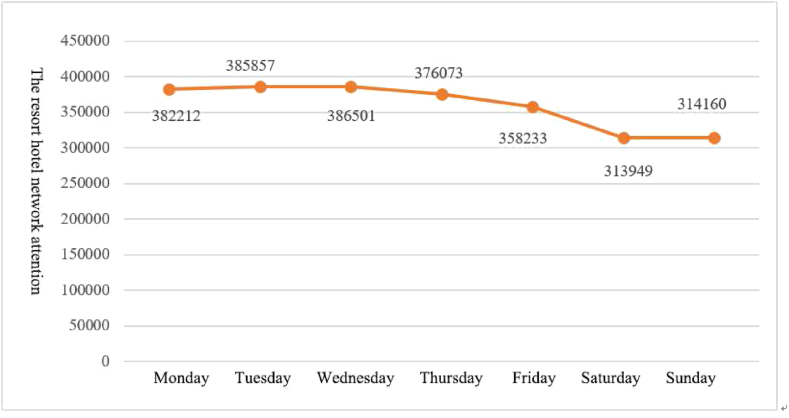
Weekly distribution characteristics of network attention to resort hotels.

The resort hotels network attention data from 2018 to 2022 were counted and summed according to the corresponding number of weeks to obtain weekly distribution characteristics. From Fig. 1, it can be seen that the online search volume of resort hotels in a week is highest on Wednesdays, with a search volume of 386,501, and relatively stable from Monday to Thursday, and there is a small decrease in the search volume from Thursday, gradually decreasing to Saturday. The lowest network attention index is on Saturday, with a volume of 313,949, and there is a small increase from Sunday. Compared with traditional city hotels, resort hotels have a relatively small number of rooms, and certain price discounts can be obtained by booking rooms in advance through online platforms. Hence, many tourists plan their itineraries in advance. The intra-week distribution skewness index of resort hotels network attention in 2018–2022 is about −4.27, indicating that it is mainly concentrated in the first four days of a week. Thus, tourists start to collect information and plan their itineraries for weekend trips in the first few days of the week, reflecting the “foreshadowing” effect of the network attention of resort hotels.

### 3.4 Analysis of the spatial distribution characteristics of resort hotels network attention

#### 3.4.1 Analysis of inter-provincial differences in resort hotels network attention

The Baidu Index utilizes the IP addresses of online search users to ascertain the geographical origin of search information. Therefore, the Baidu Index's custom time period and regional filtering capability were utilized to examine the geographical distribution characteristics of attention towards resort hotels in the network. Table 2 clearly indicates a significant disparity in the attention index of the China resort hotels network, highlighting a noticeable regional imbalance. The overall level of this index is relatively higher in the eastern region of the country than in the western region, except for Sichuan Province. The attention level in other provinces is relatively low, with most attracting less attention than the central and eastern regions. From the national point of view, the national resort hotels network concern index shows an overall decreasing trend of “east–central–west.” The regions of Guangdong, Zhejiang, Jiangsu, Shanghai, Beijing, Shandong in the east, and Sichuan province in the west currently have the highest resort hotels network attention in China. The top ten are Guangdong, Zhejiang, Jiangsu, Shanghai, Beijing, Sichuan, Shandong, Henan, Hubei, Fujian, accounting for 58.14 % of the national resort hotels network concern index in 2018. The top ten in terms of China's resort hotels network attention in 2022 were Guangdong, Zhejiang, Jiangsu, Shanghai, Beijing, Sichuan, Shandong, Henan, Hubei, and Hunan, accounting for 63.65 %. This percentage has increased since 2018, indicating that the regional differences in resort hotels network attention are increasing, and netizens are paying more attention to resort hotels in the eastern region.

**Table 2:** Spatial distribution of resort hotels network attention index, 2018–2022.

Province/Year	2018	2019	2022	2021	2022	Total	Sort by
Guangdong	48,724	48,413	42,161	39,118	36,393	214,809	1
Zhejiang	45,387	43,543	38,940	35,580	31,750	195,200	2
Jiangsu	41,841	41,302	35,077	29,713	30,873	178,806	3
Shanghai	42,359	41,583	32,075	30,296	27,917	174,230	4
Beijing	41,873	39,248	29,402	27,720	25,427	163,670	5
Sichuan	34,171	34,645	30,456	23,076	22,979	145,327	6
Shandong	31,489	29,952	26,112	22,850	21,240	131,643	7
Henan	27,895	29,980	22,030	17,101	16,730	113,736	8
Hubei	25,555	25,867	18,895	15,757	15,725	101,799	9
Fujian	26,844	25,817	17,819	13,569	12,681	96,730	10
Hebei	21,690	24,029	21,092	16,011	12,344	95,166	11
Hunan	25,918	22,743	18,121	14,127	12,708	93,617	12
Anhui	21,076	22,084	17,510	16,116	10,547	87,333	13
Liaoning	21,433	21,951	15,914	10,944	11,696	81,938	14
Yunnan	21,565	18,296	14,325	10,817	11,031	76,034	15
Chongqing	18,531	19,016	13,989	11,892	10,355	73,783	16
Shaanxi	18,241	17,531	12,194	9836	9155	66,957	17
Guangxi	17,829	17,596	13,326	9153	8694	66,598	18
Jiangxi	15,484	16,515	13,884	10,786	9280	65,949	19
Guizhou	12,376	11,228	9333	7076	5948	45,961	20
Tianjin	12,510	12,292	6327	6216	5180	42,525	21
Shanxi	10,920	12,144	6974	6317	5823	42,178	22
Hainan	9399	8501	5857	3847	4818	32,422	23
Heilongjiang	8690	8808	6506	3241	4573	31,818	24
Jilin	6848	7889	4984	3725	3802	27,248	25
Inner Mongolia	5294	6887	4202	2647	3396	22,426	26
Gansu	4960	4827	3849	1679	5206	20,521	27
Xinjiang	8608	4607	2006	2240	1940	19,401	28
Qinghai	1040	686	920	515	752	3913	29
Ningxia	728	802	684	1028	514	3756	30
Tibet	399	290	230	344	228	1491	31

According to the results of each data point in Table 3, the geographic concentration index of China's resort hotels network attention increased slightly, from 29.1210 to 29.4511, in 2018–2022. This lack of significant change indicates that the resort hotels network attention is relatively dispersed in spatial distribution. The change of H is small in all five years, being 0.0848 in 2018 and 0.0858 in 2022. This insignificant change indicates that the distribution of resort hotels network attention is relatively scattered in space, and the degree of concentration is low. The CV of resort hotels network attention was 0.1328 in 2018 and increased to 0.1716 in 2022, with a less significant increase. It indicates that the regional difference of resort hotels network attention has not much changed. 2018–2022 resort hotels network attention of P is between 1.0 and 1.1, The index indicates that resort hotels network attention ranked the first and second region, the gap between the two is small, in five years the network attention distribution is more balanced. There is no significant change.

**Table 3:** Spatial distribution characteristics index of resort hotels network attention, 2018–2022.

Various indices				
Year	Geographical concentration index G	Herfindahl Index H	Primacy P	Coefficient of variation CV
2018	29.1210	0.0848	1.0812	0.1328
2019	29.1229	0.0848	1.0860	0.1333
2020	29.1100	0.0847	1.0642	0.1297
2021	29.1100	0.0867	1.0100	0.2021
2022	29.4511	0.0858	1.0666	0.1716

#### 3.4.2 Analysis of regional differences in the attention of resort hotels networks

Further, statistical used to analyses of resort hotels network attention in different geographic regions in China were conducted, and the G, CV, H, and P among different geographic regions were calculated. From Table 4, it can be seen that of the geographical regions, the highest total value of resort hotels network attention is in East China, and the least attention is in Northeast and Northwest China. From 2018 to 2022, the values of the four indexes in East China show a small upward trend overall. The total value of network attention is the highest in East China of the seven regions: the G increases from 14.2054 to 15.6913 in 2022, and the CV increases from 0.9364 to 0.9744, indicating that the network attention within East China starts to be uneven.

**Table 4:** Spatial distribution characteristic index of resort hotels network attention.

Year/Index		Region						
		East	South	Central	North	Northeast	Northwest	Southwest
2018	Attention	224,480	75,952	79,368	92,287	36,971	33,577	87,042
	G	14.2054	8.3738	7.2827	7.9842	3.8305	3.3048	7.3284
	H	0.1271	0.014	0.0158	0.0214	0.0034	0.0028	0.0191
	P	1.0715	2.7329	1.0763	1.9305	2.4664	2.1191	1.5846
	CV	0.9364	0.7044	0.7949	0.8811	0.6714	1.0169	1.0861
2022	Attention	144,288	49,905	45,163	52,170	20,071	17,567	50,541
	G	15.6913	9.9356	6.9112	7.7733	3.4556	2.8304	7.4134
	H	0.1444	0.0173	0.0141	0.0189	0.0028	0.0021	0.0177
	P	1.0284	4.1860	1.0639	2.0599	2.5576	1.7585	2.0831
	CV	0.9744	0.7676	0.7780	0.9087	1.0145	1.1581	0.6708

All four indicators in South China increased between 2018 and 2022, with the G increasing from 8.3738 in 2018 to 9.9356 in 2022, and P increasing from 2.7329 in 2018 to 4.1860 in 2022, indicating that the concentration of network attention of resort hotels within South China is high. The difference is obvious, the highest attention is paid to Guangdong, followed by Guangxi, and the difference between Hainan and Guangdong and Guangxi is larger.

The four major indicators of Central China region show a generally decreasing trend during the period 2018–2022. The G, in particular, decreased from 7.2827 to 6.9112, indicating that the network attention of provinces within the Central China region is balanced. The highest attention is paid to Henan, followed by Hubei, the lowest is paid to Hunan, and the difference of network attention between Hunan, Hubei, and Henan is small.

Although the network attention paid to Northeast China is low, P and CV increased from 2.4664 to 0.6714 to 2.5576 and 1.0145, respectively, in 2018–2022, indicating that the difference of network attention within Northeast China is gradually increasing. The highest attention was paid to Liaoning, the lowest was to Heilongjiang, and the difference between them is large.

There is a substantial disparity in the allocation of attention to resort hotels within the North, Northwest, and Southwest regions. Moreover, there are notable variations in the attention given to resort hotels among different provinces within these regions. In 2018, the highest amount resort hotels network attention from residents of Beijing was directed at the North China region, with a network attention index of 41,873, and the lowest amount was directed at Inner Mongolia, with a network attention index of 5294. As regards resort hotels network attention directed to the Northwest region, the highest amount was directed to Shaanxi, with a network attention index of 18,241, and the lowest to Ningxia. the lowest, attention is 728, 2022 variation coefficient is 1.1581, is the highest value within the seven regions, indicating the most obvious differences within the northwest region. The network attention index directed to Sichuan was the highest within the southwest region, at 34,171, and the lowest was to Tibet at 399. The difference in network attention index directed to Chongqing, Guizhou, and Yunnan is also large.

## 4 Conclusions and discussion

### 4.1 Conclusions

From the perspective of time distribution characteristics, the national resort hotels network attention during the period 2018–2022 shows an obvious floating trend, the same in 2018–2019 shows a stable trend. Resort hotels in the years 2020–2022 were affected by the epidemic, and the network attention showed a decreasing trend. There are obvious seasonal differences in the network attention paid to China's resort hotels, and the monthly distribution is unbalanced. The network attention index of resort hotels shows a steady growth in January–April, and annual search volume peaks in July and August and then gradually declines. The peak season of annual resort hotels network search volume is March–September. The peak season of online searches for resort hotels is March–September, October–December is the flat season, and January–February is the low season. The network attention of resort hotels is mainly concentrated in the first four days of the week, reflecting the “precursor effect” of the network attention of resort hotels.

From the viewpoint of spatial distribution characteristics, there is an obvious geographical imbalance in the national resort hotels network attention index. The national resort hotels network attention index shows an overall trend of “east–central–west” decrease. The overall level of the resort hotels network attention index in the eastern region of the country is relatively high. In the western region, except Sichuan Province, and in the other provinces, the index is relatively low, and most of western region are lower than the central and eastern regions. From the regional division of resort hotels network concern index, East China has the highest concern index, and there is a serious unbalance in the distribution of resort hotels network concern index within North China, Northwest China, and Southwest China. The difference of resort hotels network concern index of the different provinces within their region is significant. Although the network concern index of Northeast China is low, the difference of network concern index within Northeast China is gradually increasing. Within South China, the resort hotels network attention is more concentrated, and the difference is obvious. The four major indicators in central China all showed a decreasing trend overall during the period 2018–2022, and the network attention of provinces within central China became more and more balanced.

### 4.2 Discussion

Through the usage of data received from search engines and the utilization of social network analysis, the purpose of this study was to investigate the utilization of data in order to expose the perspective of customers. It is a widely held belief that traditional models used to anticipate tourism demand can be enhanced by big data gathered from internet sources. These models primarily rely on traditional measurements, such as univariate analysis of visitor arrivals [26]. In order to help with tourist arrival forecasts, this study shows how well social network and semantic analysis techniques perform with large-scale data from online travel forums. Before deciding to buy a good or service, a substantial portion of customers use search engines to do internet searches. Customers' search behavior demonstrates their sincere interest. As a result, search engines are essential tools for businesses to communicate with both current and potential customers. To attain success in search engine marketing, organizations must have a thorough comprehension of consumers' web search behaviors [22,49,50].

Academics claim that each element that can impact the requirements and availability of information for individuals has an effect on online attention [51]. A number of variables, maybe including level of economic development, degree of network development, leisure time, and population size, and the spatial and temporal features of resort hotels, have been found to influence the spatial and temporal differences in network attention [52,53].

### 4.2.1 Level of economic development

The economic strength of a region will have an impact on the local consumption ability, income levels, etc. Meanwhile, the distribution of network attention is also affected by economic strength. Calculating the data of the total value of network attention and gross regional product of resort hotels in each province of China in 2022 and ranking them separately indicate that the top ten provinces in terms of total value of network attention are Guangdong, Zhejiang, Jiangsu, Shanghai, Beijing, Sichuan, Shandong, Henan, Hubei, and Fujian. Moreover, the top ten provinces in terms of gross regional product are Guangdong, Jiangsu, Shandong, Zhejiang, Henan, Sichuan, Hubei, Fujian, Hunan, and Anhui. There are 8 provinces in the top ten in both total netword attention and regional GDP rankings. The bottom ten provinces in the ranking of gross regional product are Inner Mongolia Autonomous Region, Guizhou Province, Xinjiang Uygur Autonomous Region, Tianjin City, Heilongjiang Province, Jilin Province, Gansu Province, Hainan Province, Ningxia Hui Autonomous Region, Qinghai Province, and Tibet Autonomous Region. The bottom ten provinces in the ranking of total network attention are Shanxi Province, Hainan Province, Heilongjiang Province, Jilin Province, Inner Mongolia Autonomous Region, Gansu Province, Xinjiang Uygur Autonomous Region, Qinghai Province, Ningxia Hui Autonomous Region, and Tibet Autonomous Region. There are 8 provinces in the bottom ten in both network attention value and regional GDP ranking.

Based on the aforementioned study, it is evident that there is a positive correlation between the gross product of a region and the overall value of network attention. Specifically, as the gross product increases, the total value of network attention also increases. Therefore, level of economic development is a significant determinant of the distribution of network attention across resort hotels.

### 4.2.2 Degree of network development

The quantity of Internet users will directly influence the level of attention given to the network of resort hotels. The Baidu Index measures the quantity of internet searches, which correlates with the level of network attention. Therefore, as the number of internet user increases, so does the number of searches and the level of network attention. Simultaneously, the greater degree of network development, the greater number of information channels available to tourists, the easier and simpler it is to obtain information, and the greater the resort hotels' network attention.

### 4.2.3 Leisure time

Sufficient leisure time is among the important factors influencing travelers to make travel decisions. The research of the temporal features of network attention presented earlier in this section indicates that resort hotels' network attention is mostly concentrated during the first four days of the week. This finding indicates that people use their leisure time on weekdays to collect travel information in advance for weekend outings. On the other hand, from the monthly distribution characteristics, we can see that the annual search volume of network attention peaks in July and August. This peak coincides with the traditional peak summer vacation season, when students across the country have ample and concentrated vacation time. Summer vacation is the traditional peak season for travel, and family travel and parent–child vacation are the main demands for summer travel. Thus, this finding can indicate that the distribution of network attention values of resort hotels is influenced by leisure time.

### 4.2.4 Population size

There is a clear correlation between the size of the resident population in each region and the number of people who utilize the network and tourists, as well as the amount of attention that is paid to the resort hotel network in that region.

## 5 Implication and limitations

### 5.1 Theoretical implications

One of the earliest efforts to use big data to determine the spatiotemporal characteristics and influencing aspects of network attention to resort hotels is presented in this research. This work adds to the existing literature by demonstrating the following theoretical points. First, the study found that there are obvious seasonal differences in the network attention paid to China's resort hotels, and the monthly distribution is unbalanced. which further validates the findings of previous studies in tourism [36,43,46]. Second, it's clear that the network attention index for resort hotels is geographically imbalanced, shows an overall trend of “east–central–west” decrease, it is same as other research [38,46,51]. Third, causing network attention to vary in spatial and temporal differences could consist of level of economic development, degree of network development, leisure time, and population size [7,36,38,51].

### 5.2 Practical implications

This work is a valuable contribution to the existing body of literature on data-based decision-making, particularly in relation to its implications for managers. With the improvement of economic level, the development of the network and the growth of leisure time, more and more customers choose resort hotels. High-quality resort hotels have begun to develop rapidly, becoming people's first choice for travel, and guests no longer travel simply for food, accommodation, and transportation, but also for vacation and relaxation. Hotel products need to meet the needs of more different customers, resort hotels can create “resort +” series of hotel resort products by innovating products and adding additional special experience projects, such as ice and snow, hot springs, festivals, sports, and recreation, to meet the diversified and personalized needs of leisure and vacation guests. Resort hotels should focus on the development of their own cultural products and use the local culture to attract more tourists and improve their popularity.

For the spatial and temporal characteristics of network attention, as the Internet becomes more popular, users are more inclined to use it to check relevant information before traveling, and it greatly influences people's consumption patterns. Therefore, in order to increase their market share and attract tourists, the Internet is the most important means for resort hotels to promote themselves. They can use common software products of mobile terminal for the marketing and promotion of resort hotels, such as Xiaohongshu, Jieyin, and Weibo, and cooperate with famous bloggers from all over the world to create awareness. At the same time, they can enrich their online shopping mall products on platforms such as VW Dianping, Meituan, and Ctrip to improve their conversion rates from online social platforms and promote the online marketing of their products.

In addition, there are seasonal differences in the network attention paid to China's resort hotels, so low and peak season is more obvious. In reaction to the difference between low and high seasons, on the one hand, resort hotels should adjust the guest sources through marketing strategies, strengthen marketing in holidays, weekends, and winter and summer holidays in advance, research the tourism market in advance, and expand their publicity appropriately. On the other hand, they should focus on shaping the image of the hotel, improve its visibility, focus on word of mouth, and at the same time, continuously promote product innovation to meet the needs of tourists.

The local economic situation of a resort hotels affects the network attention and thus decisions of tourists. Well-established tourism facilities can attract more tourists and gain more network attention. Therefore, firstly, regions with high popularity and a developed economy have higher network attention, and tourists are biased toward their resort hotels. Thus, resort hotels in such regions should highlight their brand, uniqueness, and high quality. Areas with lower network attention but cultural tourist attractions should improve their economic strength, improve the construction of infrastructure and quality of service, and create a unique reputation.

### 5.3 Limitations and future recommendation

While this study offers a fresh viewpoint on the examination of network attention towards resort hotels in China, it is important to acknowledge certain shortcomings that warrant additional investigation. Firstly, the Baidu index measures network attention. The Index is valuable, but it does not fully measure network attention because more individuals are using new media like short videos to look for information. Secondly, we only use a few common socioeconomic factors to correlate with network attention, and we can consider adding more factors later. Thirdly, next time, it would be use some geographical figures to show the spatial differences.

## Funding

The authors received no specific funding for this work.

## Data availability statement

The data of this study are shared on the figshare web with the https://doi.org/10.6084/m9.figshare.23943201.

## CRediT authorship contribution statement

**Huazhen Sun:** Writing – review & editing, Writing – original draft, Project administration, Methodology, Data curation, Conceptualization. **Yifeng Zhang:** Software, Formal analysis. **Weifeng Guo:** Investigation.

## Declaration of competing interest

The authors declare that they have no known competing financial interests or personal relationships that could have appeared to influence the work reported in this paper.
